# Seasonal and anthropogenic effects on niche overlap and habitat selection by sympatric bears (*Ursus arctos marsicanus*) and wolves (*Canis lupus*) in a human‐dominated landscape

**DOI:** 10.1002/ece3.70225

**Published:** 2024-10-07

**Authors:** Cecilia Parracciani, Luigi Maiorano, Paolo Ciucci

**Affiliations:** ^1^ Department of Biology and Biotechnologies “Charles Darwin” University of Rome La Sapienza Rome Italy

**Keywords:** intraguild competition, K‐select analysis, large carnivores, resource partitioning, resource selection functions

## Abstract

Interspecific interactions among species of the same guild play a critical role in shaping their realized niches, and their understanding may disclose mechanisms of coexistence. Investigating interactions among apex predators is of ecological and management interest, especially in human‐dominated landscapes where type and intensity of their interspecific competition may be affected by human interference. During 2005–2010, we investigated, by means of GPS‐telemetry, interactions between brown bears (*n* = 19) and wolves (*n* = 7) in a long‐established national park in the central Apennines, Italy, where bears and wolves have always coexisted close to humans. Based on a K‐select analysis and a randomization approach, we assessed the extent of overlap between the species' niches on a seasonal basis. Bears and wolves clearly segregated in fall but not during summer when overlap between their realized niches suggests a convergent adaptation to a seasonal peak of anthropogenic pressure. However, using multi‐species resource selection functions (RSFs) at the home range level (i.e., third‐order selection), we revealed that habitat selection by bears and wolves was reciprocally affected also when their niches overlapped, possibly disclosing mechanisms of fine‐scale resource partitioning. In early summer, bears selected areas with a high probability of resource selection by wolves, but in late summer avoided areas positively selected by wolves. On the contrary, wolves avoided areas where the probability of resource selection by bears was high, both in late summer and fall. These results indicate that bears and wolves do interact in our study area and, although the actual behavioral mechanisms are unknown, they reciprocally and asymmetrically affect their realized niche and habitat selection patterns. Further research is needed to better understand how anthropogenic factors impact intraguild interactions and what are the effects at the population and community levels.

## Graphical abstract

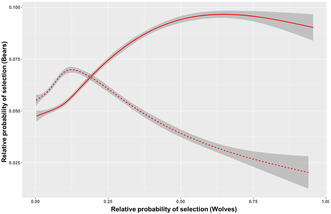

## 1 INTRODUCTION

Interspecific interactions play a critical role in shaping species' realized niche (Wisz et al., 2013), and investigating relationships between interacting species requires identifying how they use and share resources and how this relates to interspecific competition. Fine‐grain spatial and/or temporal avoidance and habitat partitioning can represent mechanisms through which two or more ecologically similar species can coexist in sympatry (Apps et al., 2006; Peters et al., 2013; Ritchie et al., 2009; Tannerfeldt et al., 2002). Accordingly, knowledge on species' ecological interactions can be gained by testing whether their distributions are correlated or independent of each other, and whether segregation occurs in terms of differential habitat use, that is to assess if habitat selection by one species is affected by the presence of the other (Darmon et al., 2012). Among others (e.g., López‐bao et al., 2016; Mattisson et al., 2011; Schmidt et al., 2009), two approaches have been traditionally used to investigate interspecific interactions. The first involves niche overlap analysis, which offers insights at the niche level as a function of species interactions within a multivariate resource space (Cozzi et al., 2012; Letten et al., 2017; Smith et al., 2018). The second involves Resource Selection Functions (RSFs; Manly et al., 2002) that, by accounting for other species' probability of selection, can be used to assess if and how resource selection is reciprocally affected (Coe et al., 2004; Gustine et al., 2006; Johnson et al., 2000).

Interactions and mechanisms that favor coexistence are relevant at the intraguild level (Elbroch et al., 2015; Ordiz et al., 2015; Peters et al., 2013). In particular, intraguild species' interactions at high trophic levels (i.e., among apex predators) can strongly affect community structure and population dynamics at lower trophic levels (i.e., mesopredators, small carnivores, herbivores, scavengers), with cascading effects through the entire ecosystem (Caro & Stoner, 2003; Ripple et al., 2014; Stahler et al., 2020). For example, complex interactions among brown and black bears, wolves, and cougars (and humans) have been found to affect community and ecosystem functioning in North America (Ballard et al., 2003; Stahler et al., 2020), similarly to the complex interactions among the rich and diversified guild of large carnivores in African systems (Caro & Stoner, 2003; Sinclair & Arcese, 1995).

Interactions among large carnivores can be either synergistic or antagonistic (sensu Tallian et al., 2022), the latter through either exploitative or interference competition (Ballard et al., 2003). While exploitative competition entails negatively affecting another species' fitness through higher efficiency in accessing a shared resource, interference competition is expressed directly through aggressive behavior and may also result in interspecific killing (Ballard et al., 2003; Palomares & Caro, 1999). In North America, for example, brown bears have been observed dominating interactions with wolves at kill sites (most often wolf kills easily usurped by bears; kleptoparasitism), even if wolves outnumbered bears (Ballard et al., 2003; Stahler et al., 2020). Through this behavioral mechanism, wolves may exert a positive effect on the bears' nutritional conditions by providing reliable food (i.e., carrion) subsidies throughout the bears' active period (Stahler et al., 2020). Differently, kleptoparasitism by bears may negatively affect wolves by limiting their access to their kills and reducing their kill rate (Tallian et al., 2017; but see Krofel et al., 2012 for the opposite effect on kill rates by lynx), with potential reflections at lower trophic levels (Tallian et al., 2022). On the contrary, wolves at homesites were found to be more successful at chasing away intruding bears, possibly attracted by meat scrapes, likely because the great investment in pups' production and development determined higher levels of persistent aggression in wolves (Ballard et al., 2003). Although rarely, wolves and bears have also been reported to occasionally kill each other (Ballard et al., 2003:Table 10.1). As witnessed by the paucity of studies on this topic, and the anecdotal nature of the data available, interactions among large carnivores are difficult to assess experimentally, making it difficult to infer if intraguild competition observed at the individual level reflects in population responses (Ordiz et al., 2015; Stahler et al., 2020). In this perspective, more recent studies at the biogeographic scale revealed that bear density plays a negative effect on the probability of territory establishment in the expanding Scandinavian wolf population (Ordiz et al., 2015) and that, where sympatric, brown bears and wolves segregate more than expected by chances when selecting the habitat within the home range (Milleret et al., 2018).

In the human‐modified landscape of Europe, various forms of anthropogenic interference are expected to play an overwhelming role in affecting the dynamics of intraguild competition among large carnivores (Apps et al., 2006; Ordiz et al., 2015; Smith et al., 2017, 2018). For instance, human activity can reduce the spatial and temporal avoidance of heterospecific competitors as a means of avoiding human disturbance (Smith et al., 2017, 2018). Although large carnivores in Europe are rebounding after centuries of persecution, currently featuring stable or expanding populations (Chapron et al., 2014), the studies addressing the interaction among them are still rare and limited to a few countries (Krofel et al., 2012; Milleret et al., 2018; Ordiz et al., 2015; Ordiz, Milleret et al., 2020; Ordiz, Uzal et al., 2020; Schmidt et al., 2009; Tallian et al., 2017, 2022; Wikenros et al., 2010). This knowledge, however, is important to fully understand to what extent the potential ecological effects of apex predators through communities and ecosystems may be altered by anthropogenic interference (Dorresteijn et al., 2015; Kuijper et al., 2016; Ordiz et al., 2021).

We investigated the ecological interactions between brown bears and wolves in a long‐established national park (Abruzzo, Lazio and Molise National Park; PNALM) in central Italy, where both species have coexisted since historical times. The PNALM, established back in 1923, hosts the last remnant of the Apennine brown bear population, a relict and highly imperiled bear population facing high and prolonged extinction risks due to its long isolation from other European bear populations, its low genetic variability, and accumulated deleterious mutations (Benazzo et al., 2017; Ciucci & Boitani, 2008). Along with bears in the Cantabrian mountains (NW Spain), Apennine bears are the sole autochthonous small bear population still remaining in southern Europe (Penteriani et al., 2020), hence of particular conservation value. The PNALM also represented a relevant historical stronghold for Italian wolves when the population faced the highest extinction risk in the late 1960s (Zimen & Boitani, 1975). Currently, while wolves thrive locally on a rich and diversified wild prey community, being also largely subsidized by anthropogenic food sources (Ciucci et al., 2020), bears have a predominantly vegetarian diet, with meat consumption limited to scavenging carrions of large wild and domestic ungulates, mostly during the spring following den emergence (Careddu et al., 2021; Ciucci et al., 2014). Although behavioral interactions between brown bears and wolves in the PNALM are scarcely observed, extensive dietary analyses on both wolves (Ciucci et al., 2020) and bears (Ciucci et al., 2014) indicate lack of interspecific predation and/or scavenging, while anecdotal observations indicate that brown bears can usurp wolf kills or displace wolves feeding at a carcass. The long coexistence between Apennine brown bears and wolves in the PNALM offers a unique opportunity to assess their interactions in an area historically shaped by humans. Enhancing our understanding of their intraguild ecology would also allow informing ecologically more sound conservation strategies integrated at the intraguild level (Ciucci & Boitani, 2008).

We used Global Positioning System (GPS) locations collected from bears and wolves in the PNALM to investigate their niche segregation and resource partitioning on a seasonal basis. Following Milleret et al. (2018), we adopted niche overlap analysis to assess niche interaction between bears and wolves at the home range scale. We then complemented our analysis using a multi‐species RSF approach (Johnson et al., 2000) to estimate the nature and extent of resource selection by the two species. Specifically, by accounting for seasonal variation, our objectives were to (i) estimate the degree of overlap between the realized niches of sympatric bears and wolves, and (ii) assess interspecific effects on habitat selection by both species. We hypothesized that, depending on the season, bears' and wolves' realized niches would segregate, and that resource selection by bears would negatively affect resource selection by wolves (H1). This is expected as brown bears, due to their larger size, are drivers of intraguild interactions, with negative effects on wolves through mechanisms of exploitative and interference competition (e.g., kleptoparasitism; Ballard et al., 2003). We also hypothesized that niche segregation and resource partitioning between bears and wolves would decrease at increasing levels of human activity (i.e., during summer and daylight hours), when both species are expected to converge on the habitat features that most ensure segregation from humans as a risk avoidance strategy (H2).

## 2 MATERIALS AND METHODS

### 2.1 Study area

**FIGURE 1:**
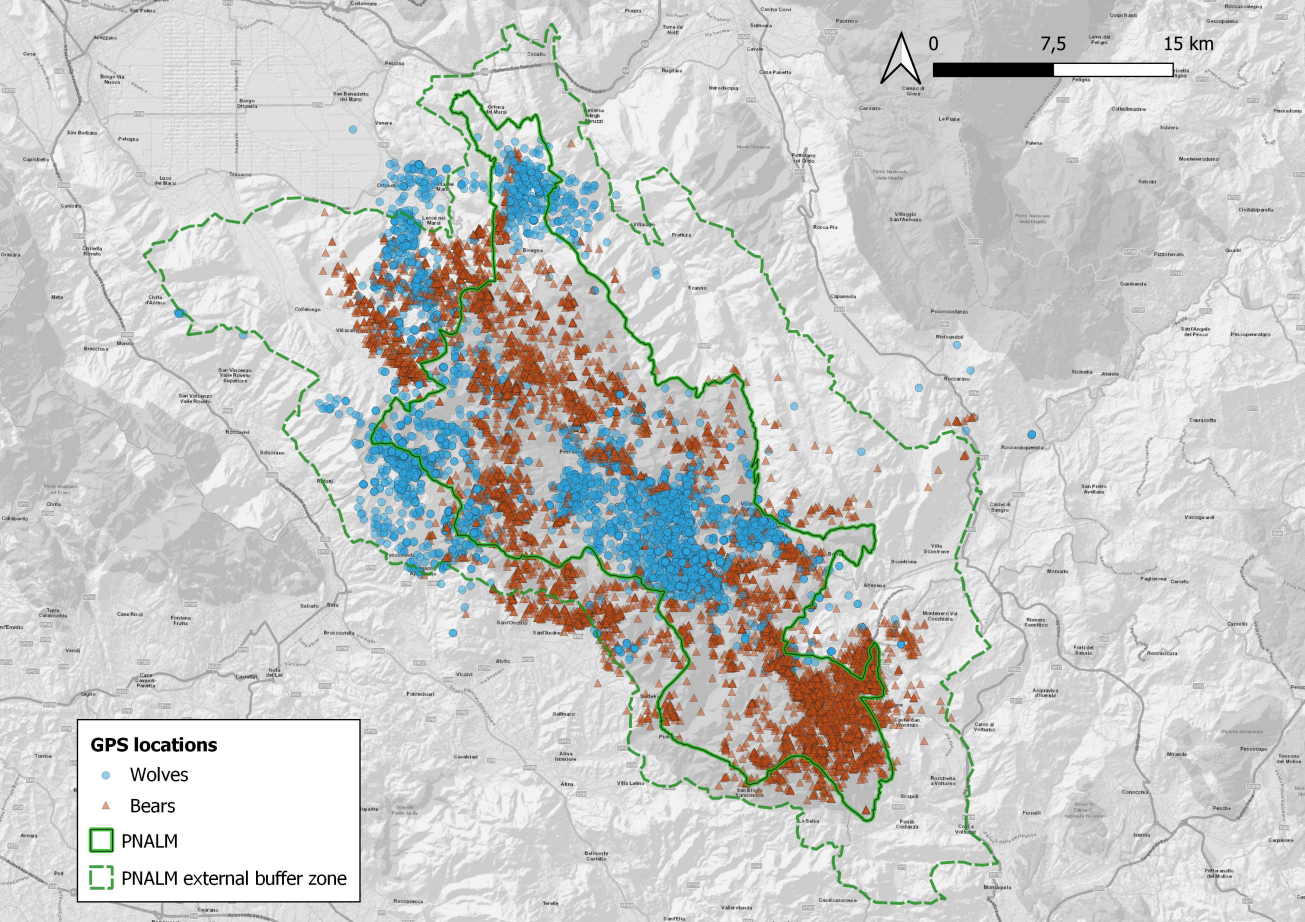
Global Positioning System (GPS) locations of adult Apennine brown bears (*n* = 10,365) and wolves (*n* = 4937) in the Abruzzo Lazio and Molise National Park, central Italy (2005–2010). GPS locations were used to assess niche overlap and species interaction by means of K‐select analysis and multi‐species Resource Selection Functions, respectively.

Our 1800 km^2^ study area was in the central Apennines, Italy, comprising the Abruzzo, Lazio, and Molise National Park and adjacent areas (PNALM; Figure 1). The area is typically mountainous, with elevations ranging from 400 to 2285 m, and is characterized by a Mediterranean montane climate, with dry summers and cold, snowy winters. Average monthly temperatures range from 2°C in January to 20°C in July, with more frequent rainfall in spring and autumn; snow cover is generally present from mid‐December to March (Ciucci et al., 2015). About 60% of the study area is covered by deciduous forests, mostly beech (*Fagus sylvatica*) at higher altitudes, followed by subalpine meadows and pastures, and agricultural areas (Ciucci et al., 2014). Human density averages 14.6 inhabitants/km^2^ and road density, including unpaved roads, averages 1.1 km/km^2^ (Ciucci et al., 2015). Economic activities in the park comprise livestock husbandry, forestry, and tourism, the latter being highly concentrated from June through August.

The PNALM features a rich community of wild ungulates, including wild boar (*Sus scrofa*), red deer (*Cervus elaphus*), Apennine chamois (*Rupicapra pyrenaica ornata*), and roe deer (*Capreolus capreolus*). Both wolves and bears have always coexisted in the PNALM, currently featuring appreciably high densities (i.e., 5 wolves/100 km^2^ and 39 bears/1000 km^2^; Ciucci et al., 2015; Mancinelli et al., 2018). Bears and wolves are legally protected both within and outside the PNALM, even though human‐caused mortality (i.e., illegal and incidental) is not negligible (Ciucci et al., 2020; Falcucci et al., 2009).

### 2.2 GPS relocations of wolves and bears

For the scope of this work, we used location data collected from 19 adult bears (11 females and 8 males) and 7 wolves (3 females and 4 males) in the PNALM ecosystem from 2005 to 2010 (Figure 1), both equipped with Global Positioning System (GPS)‐collars (see Gervasi et al., 2012; Mancinelli et al., 2018 for further details on live‐trapping and handling). Although GPS locations were originally acquired at different rates depending on the species and season, we standardized the acquisition rate to 1 location every 3 h across species and individuals for the scope of this study. Overall, we used 10,365 GPS locations from 29 bear‐years and 4937 GPS locations from 8 wolf pack‐years (Table A1).

### 2.3 Modeling

We used two complementary approaches to assess niche overlap and habitat selection by bears and wolves at the third order of selection (i.e., within the home range; Johnson's, 1980). First, we used K‐select analysis to quantify the degree of niche overlap between the two species (Milleret et al., 2018). Second, we used RSFs to investigate if habitat selection (i.e., the relative probability of resource selection; Johnson et al., 2000) by one species was affected by the other and vice versa. While K‐select focuses on the marginality concept and allows estimating the overlap in the realized niche (Milleret et al., 2018), RSFs evaluate the effects that the interaction between the two species might have in shaping their habitat selection. K‐select detects cumulative, multidimensional changes at the niche level (Milleret et al., 2018), while RSFs supplement these results with information at a finer behavioral scale (i.e., resource selection) (Coe et al., 2004; Gustine et al., 2006; Johnson et al., 2000). For example, it could be theoretically possible to estimate an overlapping niche between the two species with K‐select, indicating they use the same resources, while revealing a negative interaction through RSFs, the latter indicating fine‐scale resource partitioning. To account for seasonality in the analysis, we considered four seasons based on the wolves' and bears' annual cycles, the phenology of bear key foods (Ciucci et al., 2014), and human activity in the area: spring (March–May); early summer (June–July); late summer (August–September); autumn (October–December).

#### 2.3.1 Environmental and anthropogenic variables

Focusing on known habitat relationships of both species in the study area (Maiorano et al., 2015; Mancinelli et al., 2019), we originally considered 16 predictor variables (Table 1). To account for topographic variables, we used a Digital Elevation Model (DEM; original resolution 20 × 20 m), provided by the Italian Military Geographic Institute, from which we derived altitude, slope (mean and standard deviation), a Terrain Ruggedness Index (TRI; Riley et al., 1999), and hillshade (QGIS hillshade, GDAL plugin). For land cover data, we obtained the regional Corine Land Cover layers (CLC; scale 1:10,000) from each administrative region (Lazio, Abruzzo, and Molise), and we aggregated the original land cover classes into four categories. From the same land cover data, we also calculated the distance to forest edges, with negative values inside the forest and positive outside (Falcucci et al., 2009). Canopy cover, tree density, and tree basal area were obtained by Falco (2021), originally derived from the Tree Cover Density layers of the Copernicus Land Monitoring Service (https://land.copernicus.eu/en/products/high‐resolution‐layer‐tree‐cover‐density). We also considered anthropogenic variables, including the Euclidean distance to settlements (National Institute of Statistics, updated 2011; https://www.istat.it/notizia/basi‐territoriali‐e‐variabili‐censuarie/) and to both primary and secondary roads (De Agostini, GeoNext, and TeleAtlas databases, updated to 2003). All the GIS layers used in the analysis were resampled to a 30 × 30 m cell size (i.e., resource units). To obtain a better approximation of the composition of the environment around bears' and wolves' relocations (Falcucci et al., 2009), we used a 400‐m radius moving window (Falcucci et al., 2009) and a map‐algebra function to transform categorical into continuous variables (Maiorano et al., 2019). To avoid problems due to collinearity in single‐ and multi‐species RSFs, we used Pearson correlation (*r* ≥ 0.5) and Variance Inflation Factors (VIF > 5) discarding correlated variables (Zuur et al., 2009). Out of 16 original variables, after checking for collinearity we retained 11 variables, and the same were also used in the K‐select analysis to facilitate comparison with the multi‐species RSFs (Table 1). Finally, to allow comparison of covariates' effects and to improve model convergence, we standardized the selected variables by subtracting the mean and dividing by the standard deviation (Zuur et al., 2009).

**TABLE 1:** Covariates considered to assess niche overlap between Apennine brown bears and wolves and their interaction effect on habitat selection in the Abruzzo Lazio and Molise National Park, central Italy (2005–2010).

Variable			
Type	Description	Units of measure	Code
Land cover	Shrubland^a^	%	Shrub
	Pastures and grasslands^a^	%	PastGrass
	Non‐vegetated rocky areas^a^	%	NoVeg
	Agricultural areas^a^	%	Agri
Forest‐related	Distance to forest edges^a^, ^b^	m	DistForEdge
	Average density of trees in beech forest^a^	n° trees/hectare	TDBeech
	Average density of trees in oak and hop‐hornbeam forest^a^	n° trees/hectare	TDOak
	Tree Cover Density	%	TCD
	Tree basal area	average tree basal area/hectare	TBA
Anthropogenic	Distance from urban centers and primary roads^a^	m	DistSettlR1
	Distance from secondary roads^a^	m	DistR2
Topographic	Altitude	m	Elev
	Average slope	degrees	SlopeM
	Standard deviation of slope	degrees	SlopeSD
	Terrain Ruggedness Index^a^	‐	TRI
	Hillshade^a^	‐	Hillsh

*Note*: Due to collinearity in multi‐species RSFs, we did not retain all covariates in the final analyses.

^a^ Variables retained in analyses after checking for collinearity.

^b^ Positive values outside and negative values inside the forest.

#### 2.3.2 K‐select analysis

Following Milleret et al. (2018), we estimated the extent of niche overlap between bears and wolves using K‐select analysis (Calenge et al., 2005). According to a use vs availability design at the individual level, we defined, for each individual and on a seasonal basis, the habitat available as the resources comprised within the Minimum Convex Polygon (100% MCP), whereas resources used were represented by individual GPS locations. Specifically, by calculating for each species the marginality scores (the differences between the average available and used habitat) of the GPS locations obtained for each individual on each dimension (axis) of the K‐select analysis, we obtained for each season the relative distribution of individuals within the ecological space generated by K‐select. Each habitat variable defines one dimension in the ecological space, and the vector of the differences between average available and used habitat (marginality) quantifies the strength and direction of the selection (Calenge et al., 2005). Therefore, the direction indicates the variables selected, and the marginality score indicates the intensity of the use (Milleret et al., 2018).

To calculate an index of overlap between the two species' distributions within the space defined by the first two axes of the K‐select, we then used a density function based on a non‐parametric Gaussian kernel (Geange et al., 2011; Mouillot et al., 2005). The overlap index ranges from 0 (complete segregation) to 1 (complete overlap). We also accounted for circadian effects, thus obtaining three indices of overlap for each of the two axes: one referred to daylight hours, one to the night (both calculated using the solar angle as a function of position and time, *OCE* R‐package; Kelley, 2018), and a third referred to the whole day. Subsequently, for each season and for each of the indices of overlap, we calculated the average overlap index ${\bar{S}}_{\mathrm{ij}}$ between the species *i* and *j*, weighed by the eigenvalues (λ) of each axis (*t*) of the K‐select, as defined in Equation 1:

(1) $${\bar{S}}_{\mathrm{ij}}=\frac{\sum_{t}^{n}S_{{\mathrm{ij}}_{t}}\lambda_{t}}{\sum_{t}^{n}\lambda_{t}}$$

For each season, we then obtained three average indices of overlap, ${\bar{S}}_{\mathrm{ij}}$, that is ${\bar{S}}_{\mathrm{ij}}$ daylight (*D*); ${\bar{S}}_{\mathrm{ij}}$ night (*N*); ${\bar{S}}_{\mathrm{ij}}$ whole day (24 h).

To test whether bears and wolves overlapped their niche more than expected by chance, we implemented a null model using the R package adehabitatLT (Calenge, 2006). Specifically, for each species we randomly rotated (*n* = 250) the observed GPS trajectories of each individual around their centroid, where individual trajectories had constant time lag between successive relocations (i.e., regular trajectories; Calenge et al., 2009). To account for missing locations (i.e., gaps between consecutive steps ≥24 h), we subdivided the complete trajectory of each individual into smaller trajectories (bursts). The simulation method above, reflecting the autocorrelation between successive relocations along a trajectory, simulates random habitat use (null model) linking all the random permutations of the frequencies of use to the properties of the individual trajectory (Calenge et al., 2005; Martin et al., 2008). For each of the 250 simulated trajectories, we estimated the indices of overlap described above to obtain randomly generated reference distributions of the expected indices of overlap under the null hypothesis (i.e., habitat randomly used). For each species, season, and circadian period, we then compared the observed indices of overlap with the simulated distribution using a two‐tailed test at α = .05. It is important to stress that the significance of the observed overlap value is independent of its absolute value (randomization test). Indeed, the niche overlap index varies between 0 and 1, but the significance of the observed value is estimated exclusively with respect to the seasonal distribution of the simulated indices.

#### 2.3.3 Resource selection functions

To investigate if the relative probability of resource selection by one species affected extent and direction of habitat selection by the other, we developed seasonal, multi‐species RSFs whose covariates, in addition to environmental and anthropogenic variables, included the probability of resource selection by the other species, previously estimated by single‐species RSFs (i.e., interspecific predictor; Gustine et al., 2006; Johnson et al., 2000; Leblond et al., 2016). For both single‐ and multi‐species RSFs, we assessed collinearity among model covariates prior to model development (Table A2). We used the same use vs availability design adopted for the K‐select analysis (see above), except we randomly sampled seasonal 100% MCPs at a density of 10 locations/km^2^ to represent availability.

For each species and season, we developed both single‐ and multi‐species RSFs using mixed effects regression models (GLMM) with the lme4 R‐package (Bates et al., 2015) and a logit‐link function with individual ID (or pack‐ID for wolves) as a random intercept factor (Gillies et al., 2006). We did not discriminate between daylight and night in RSFs to simplify the models and because the K‐select analysis did not reveal marked circadian effects (see 3. Results). Starting from the full model, we used model dredging (dredge function in MuMIn R‐package; Barton, 2020) to compare alternative models using the sample‐size corrected Akaike's Information Criterion (AIC_*c*_). We then adopted multimodel inference by averaging coefficients based on model weights, limited to models whose ΔAICc score was ≤10 from the most supported model (Burnham & Anderson, 2002). Using the Ecospat R‐package (Broennimann et al., 2020), we finally assessed the predictive power of the final multi‐species RSFs through the Boyce index, which ranges between −1 and +1 with values close to 1 indicating a high predictive power (Boyce et al., 2002). The validation of each model was repeated 100 times subdividing a training (90%) and a validation (10%) dataset, and by averaging values.

## 3 RESULTS

### 3.1 Niche overlap

**FIGURE 2:**
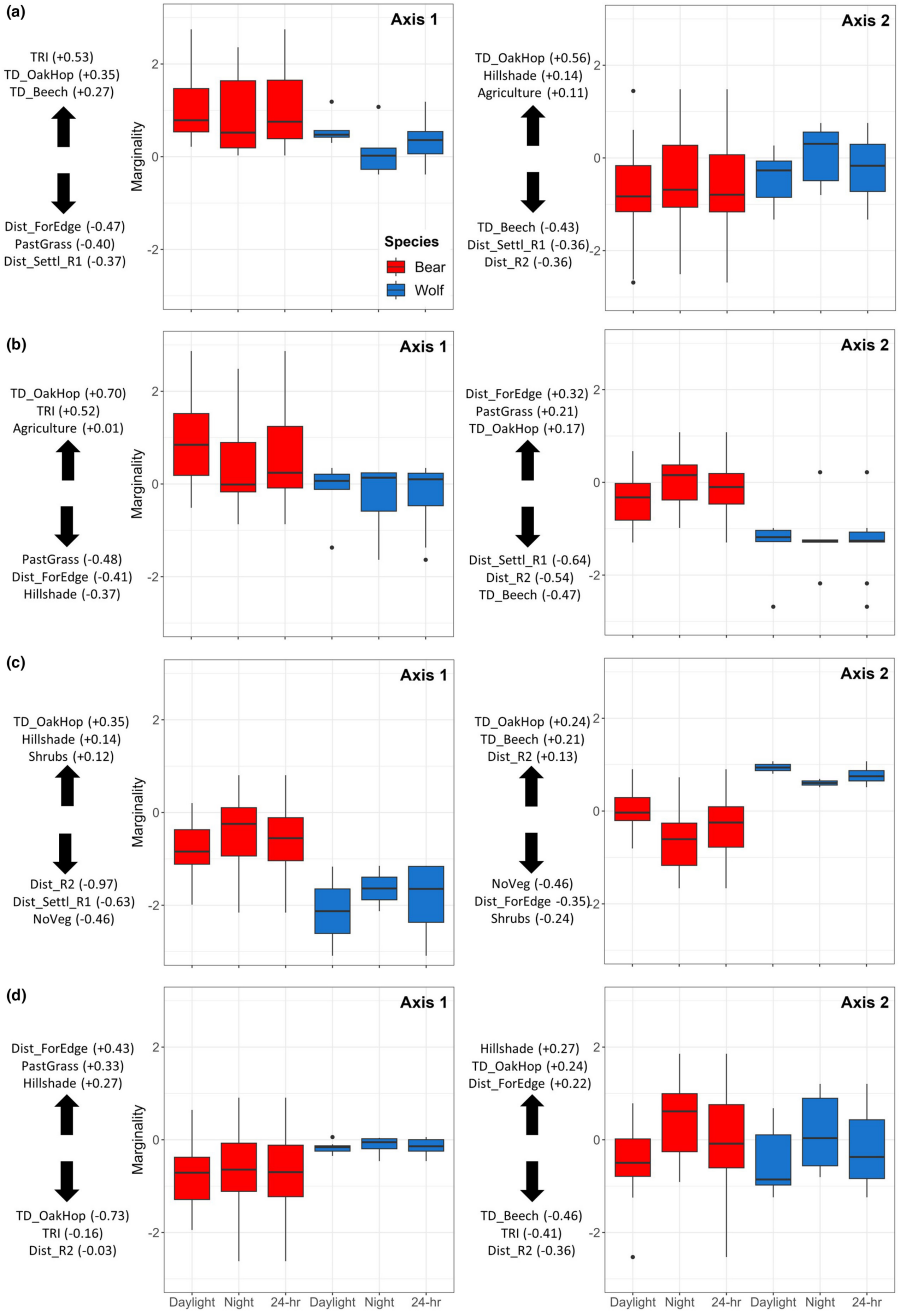
Box‐plot of the K‐select analysis for habitat selection by bears (red) and wolves (blue) in the Abruzzo Lazio and Molise National Park, central Italy (2005–2010). (a) Spring, (b) Early Summer, (c) Late Summer, and (d) Autumn. Box‐plots show marginality scores per species for daylight hours, night, and across the 24 h, for the first 2 axes of the K‐select. The three variables contributing the most on each axis are shown on the left side of each panel, with positive values above the arrow and negative values below the arrow. The scores of the three variables contributing the most are represented in brackets.

Depending on the season, the first two axes of the K‐select explained 68–76% of the marginality and were retained for the analysis. In spring, we revealed strong similarities between bears and wolves, as both species were characterized by positive scores on the first axis, indicating selection for rugged terrain, high tree density, and areas further from settlements and roads, while avoiding open areas (Figure 2a). However, bears and wolves did segregate in spring during daylight hours (${\bar{S}}_{\mathrm{ij}}$ daylight = 0.53; *p* ≤ .05; Table 2), with bears selecting rugged terrain and tree density more than wolves (Figure 2a). We revealed similar tendencies in early summer, with no apparent niche segregation (Table 2) and habitat selection patterns similar to spring and comparable between bears and wolves (Figure 2b). In late summer, we also failed to detect any significant segregation between wolves and bears (Table 2), with habitat selection mostly shaped by selection of areas further from human infrastructures and avoidance of highly exposed sites (Figure 2c). In autumn, bears and wolves segregated their niches more than expected by chance, regardless of the time of day (0.48 ≤ ${\bar{\;S}}_{\mathrm{ij}}$ ≤ 0.57; 0.01 ≤ *p* ≤ .05; Table 2). In this season, even though both species featured negative marginal scores (first axis), bears selected high‐density oak forests and rugged terrain, while avoiding open areas far from the forest edge and proximity to secondary roads, proportionally more than wolves (Figure 2d).

**TABLE 2:** Seasonal indices of niche overlap between Apennine brown bears and wolves (${\bar{S}}_{\mathrm{ij}}$) calculated by K‐select analysis.

Season	${\bar{S}}_{\mathrm{ij}}$		
	Daylight	Night	Whole day
Spring	0.53^a^	0.63	0.70
Early summer	0.31	0.43	0.44
Late summer	0.42	0.29	0.43
Autumn	0.48^b^	0.57^a^	0.55^b^

*Note*: The index varies between 0 (complete segregation) and 1 (complete overlap). The null hypothesis is that the two species have a totally overlapping niche (${\bar{S}}_{\mathrm{ij}}$ = 1). Significant *p*‐values indicate that the overlap between the realized niche of the two species is significantly lower (i.e., habitat segregation) than it would be expected by chance alone (i.e., null model). Data refer to Global Positioning System locations of bears (*n* = 10,365) and wolves (*n* = 4937) in the Abruzzo Lazio and Molise National Park, central Italy (2005–2010).

^a^ *p* ≤ .05.

^b^ *p* ≤ .01.

### 3.2 Resource selection functions

**FIGURE 3:**
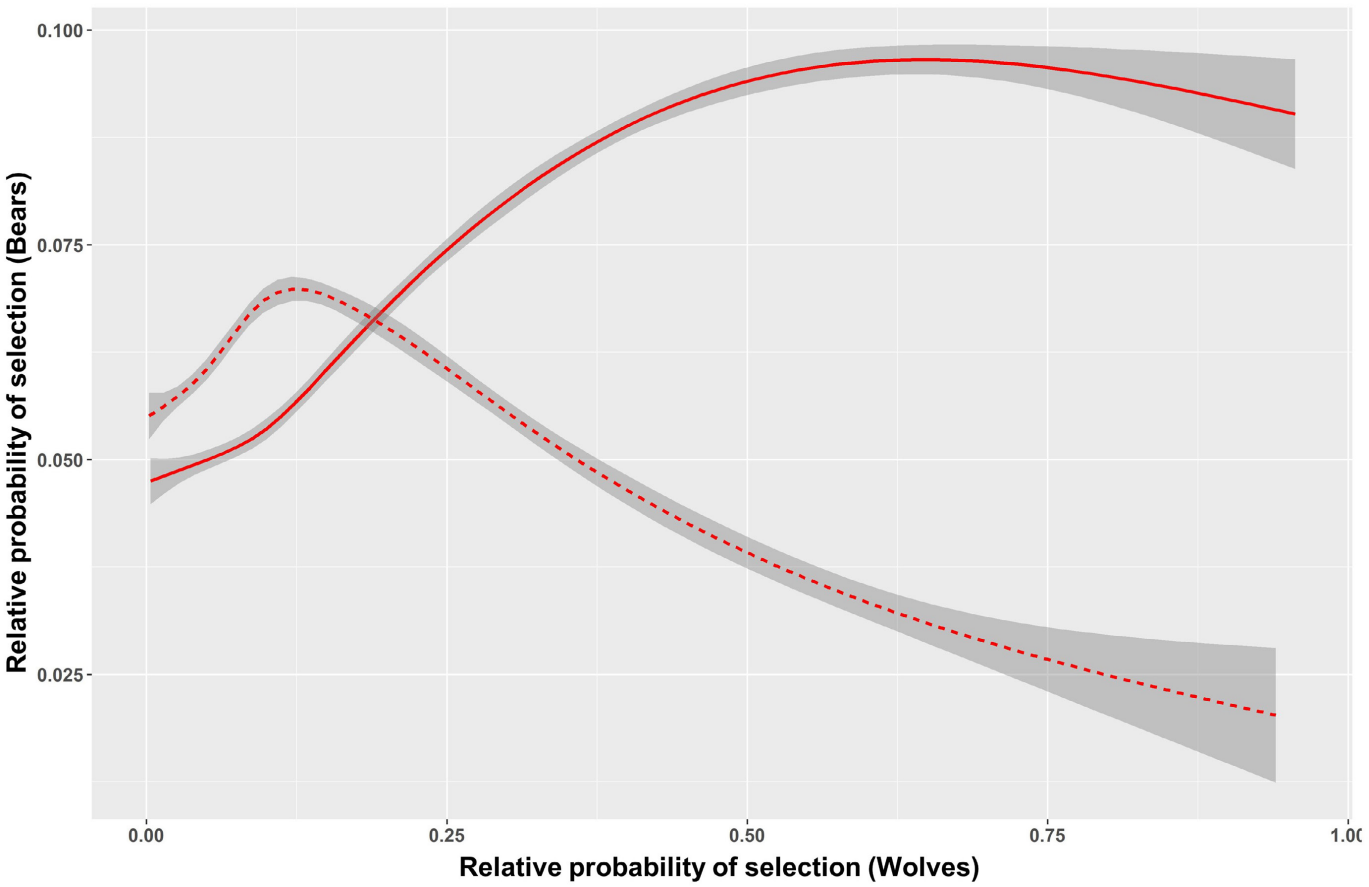
Relative probability of resource selection by bears as a function of resource selection by wolves based on multi‐species Resource Selection Functions. Solid line: Early summer; dashed line: Late summer. Gray areas represent 95% confidence intervals.

**FIGURE 4:**
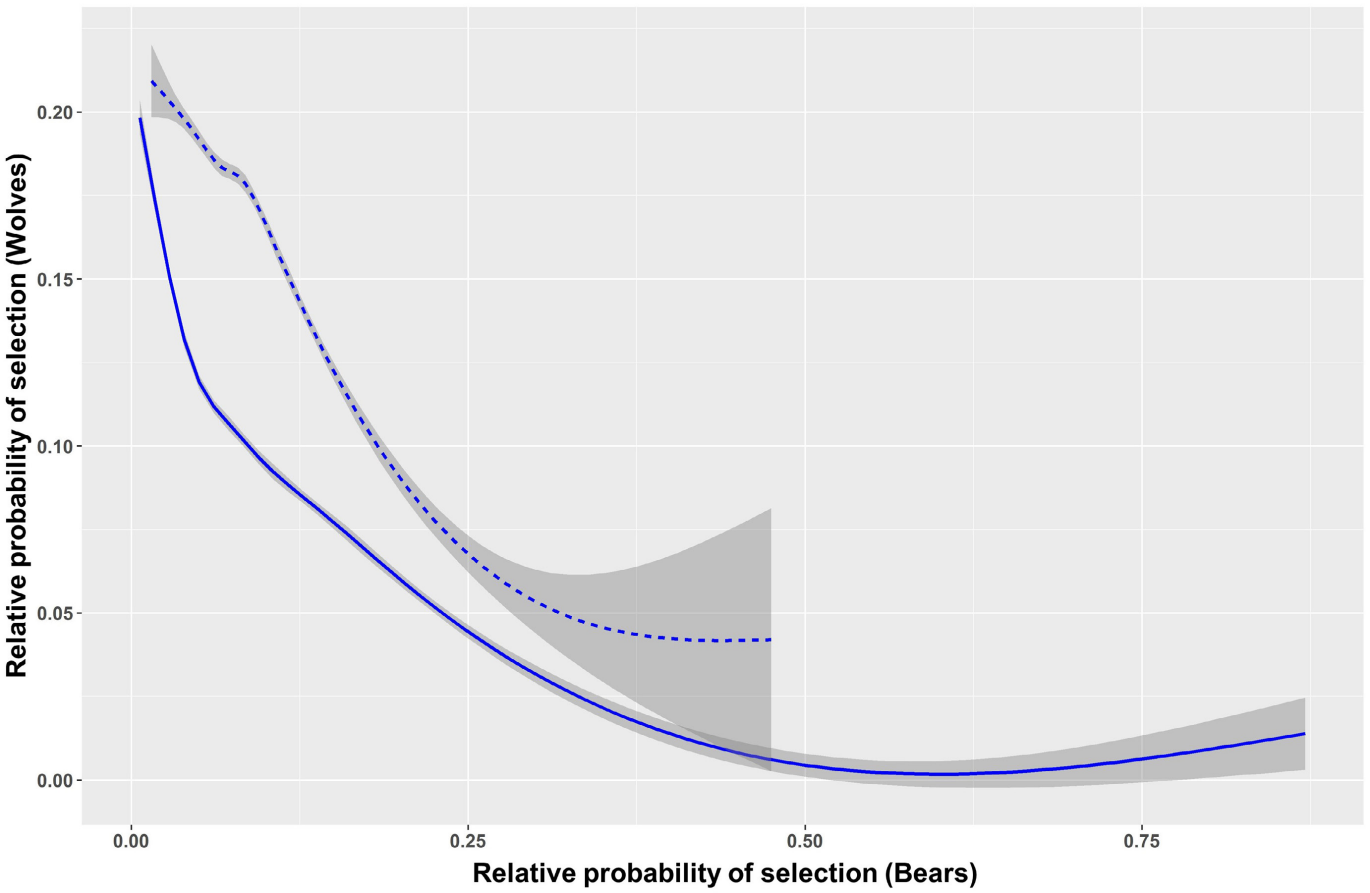
Relative probability of resource selection by wolves as a function of resource selection by bears based on multi‐species Resource Selection Functions. Solid line: Late summer; dashed line: Autumn. Gray areas represent 95% confidence intervals.

The best selected models for all seasonal, multi‐species RSFs showed good predictive power, both for bears (0.67 ± 0.18 ≤ Boyce index ≤0.81 ± 0.13) and more so for wolves (0.81 ± 0.12 ≤ Boyce index ≤0.91 ± 0.11). Notably, all the selected seasonal, multi‐species RSFs models consistently included the interspecific predictor (Tables A3 and A4). For bears, the wolf interaction term was significant in early and late summer (Table 3). In early summer, bears tended to select areas featuring high probability of resource selection by wolves, but in late summer they did just the opposite (Figure 3). Conversely, for wolves the bear interaction term was significant in late summer and autumn (Table 3). In both seasons, wolves consistently tended to avoid areas of high probability of resource selection by bears, an effect notably stronger in fall than in late summer (Figure 4).

**TABLE 3:** Coefficients of multi‐species Resource Selection Functions and their 95% confidence intervals (95% CI) to investigate seasonal habitat selection by Apennine brown bears and wolves at the third‐order selection in the Abruzzo Lazio and Molise National Park, central Italy (2005–2010).

		Spring				Early summer				Late summer				Autumn			
Species	Variable	β	SE	95% CI		β	SE	95% CI		β	SE	95% CI		β	SE	95% CI	
				Lower	Upper			Lower	Upper			Lower	Upper			Lower	Upper
Bear	(Intercept)	−2.96	0.28	−3.5	−2.4	−2.59	0.20	−2.98	−2.21	−2.53	0.23	−2.99	−2.07	−2.31	0.22	−2.74	−1.88
	Agri	−.16	0.06	−0.28	−0.04	.19	0.04	0.12	0.26	−.06	0.08	−0.23	0.10	.17	0.03	0.11	0.23
	NoVeg	−.46	0.06	−0.59	−0.33	.29	0.03	0.22	0.35	.49	0.04	0.42	0.56	.04	0.07	−0.09	0.17
	PastGrass	−.75	0.07	−0.88	−0.61	−.05	0.06	−0.16	0.06	.27	0.05	0.17	0.37	−.27	0.06	−0.38	−0.16
	Shrub	−.04	0.04	−0.12	0.04	.03	0.03	−0.04	0.10	.28	0.03	0.23	0.33	−.09	0.03	−0.16	−0.03
	DistForEdge	.25	0.06	0.13	0.36	.17	0.04	0.08	0.25	−.07	0.05	−0.16	0.03	.01	0.04	−0.06	0.08
	DistR2	.28	0.04	0.20	0.37	.05	0.04	−0.02	0.12	.43	0.03	0.37	0.49	.19	0.04	0.11	0.27
	DistSettlR1	−.11	0.05	−0.21	−0.00	.01	0.03	−0.05	0.08	.27	0.05	0.17	0.38	−.21	0.04	−0.29	−0.14
	TDBeech	.23	0.07	0.09	0.37	.43	0.06	0.32	0.54	.68	0.06	0.57	0.79	.08	0.06	−0.05	0.21
	TDOak	.09	0.06	−0.04	0.21	.65	0.04	0.56	0.74	.23	0.05	0.13	0.33	.28	0.04	0.20	0.35
	Hillsh	−.12	0.03	−0.17	−0.07	−.16	0.02	−0.20	−0.12	.01	0.02	−0.03	0.05	−.06	0.03	−0.11	0.00
	TRI	.39	0.03	0.33	0.44	.33	0.04	0.26	0.40	−.08	0.03	−0.15	−0.02	.22	0.04	0.15	0.29
	WolfRSF	.02	0.05	−0.07	0.12	.23	0.04	0.15	0.32	−.26	0.04	−0.33	−0.19	.08	0.08	−0.07	0.23
Wolf	(Intercept)	−2.26	0.45	−3.14	−1.38	−2.11	0.19	−2.49	−1.72	−2.6	0.28	−3.15	−2.07	−1.63	0.31	−2.24	−1.02
	Agri	−.01	0.03	−0.08	0.05	−.23	0.12	−0.46	0.01	.03	0.07	−0.09	0.15	.19	0.04	0.12	0.26
	NoVeg	−.67	0.11	−0.88	−0.45	−.19	0.06	−0.30	−0.08	.54	0.08	0.39	0.69	−.22	0.04	−0.30	−0.13
	PastGrass	.00	0.03	−0.05	0.05	−.18	0.08	−0.34	−0.01	−.34	0.08	−0.49	−0.19	−.23	0.04	−0.32	−0.15
	Shrub	−.04	0.04	−0.12	0.05	−.27	0.07	−0.40	−0.14	−.13	0.08	−0.29	0.04	.01	0.02	−0.03	0.04
	DistForEdge	−.40	0.04	−0.48	−0.31	−.61	0.06	−0.72	−0.50	−.31	0.07	−0.43	−0.18	−.14	0.04	−0.22	−0.06
	DistR2	.38	0.04	0.30	0.45	.47	0.05	0.38	0.57	.99	0.07	0.85	1.12	.28	0.04	0.20	0.36
	DistSettlR1	−.44	0.05	−0.54	−0.34	.74	0.06	0.62	0.86	.84	0.06	0.73	0.95	.01	0.03	−0.05	0.07
	TDBeech	.15	0.05	0.06	0.25	.19	0.08	0.04	0.34	.37	0.10	0.18	0.55	−.02	0.03	−0.08	0.05
	TDOak	.02	0.04	−0.05	0.09	.01	0.04	−0.07	0.09	.20	0.08	0.04	0.36	.29	0.04	0.21	0.37
	Hillsh	.06	0.04	−0.01	0.13	.20	0.05	0.11	0.28	.43	0.05	0.33	0.53	−.10	0.03	−0.15	−0.04
	TRI	−.02	0.04	−0.09	0.05	−.71	0.06	−0.83	−0.59	−.58	0.06	−0.69	−0.47	−.00	0.02	−0.05	0.04
	BearRSF	.01	0.04	−0.06	0.08	.17	0.10	−0.03	0.37	−.70	0.10	−0.90	−0.51	−.27	0.05	−0.37	−0.16

*Note*: See Table 1 for variable coding.

Complementary to K‐select, contrasting seasonal habitat selection by bears and wolves provides indications of resource partitioning between them (Table 3). In spring, while K‐select did not reveal niche segregation between bears and wolves throughout the day or during the night, multi‐species RSFs indicated that bears, compared to wolves, avoided open areas and exposed sites, and selected for terrain ruggedness. Similarly, while no segregation between the two species was apparent in early and late summer with K‐select, multi‐species RSFs indicated difference in habitat selection both in direction (early summer) and extent (late summer) (Table 3). For instance, while bears in early summer selected open areas and terrain ruggedness, were unresponsive to human infrastructures, and avoided exposed sites, wolves avoided areas with no or open vegetation, rugged terrain, proximity to roads and settlements but selected for exposed sites (Table 3). In late summer, while direction of habitat selection was remarkably similar between the two species (Table 3), bears selected, proportionally more than wolves, for tree density and shrublands, whereas wolves avoided, proportionally more than bears, areas closer to human infrastructures, further from forest edges, and selected for more rugged terrain and exposed sites (Table 3).

Interestingly, in the seasons when the interspecific coefficient was significant for one or both species, the coefficient of some of the other covariates differed between the single vs the multi‐species RSFs, both in extent and/or direction (Tables A5 and A6). For instance, in late summer, single‐species RSF revealed bears avoiding agricultural fields, highly exposed sites, and areas further from settlements; however, when accounting for the wolf interaction effect (i.e., multi‐species RSF), the former two covariates lost significance and the latter was inversely selected (i.e., bear selected areas further from settlements) (Table A5). Similarly, according to the single‐species RSF, wolves in late summer avoided non‐vegetated areas and shrublands and were unresponsive to tree density of both beech and oak forests; however, when accounting for the bear interaction effect, wolves selected for non‐vegetated areas and were unresponsive to shrublands (Table A6).

## 4 DISCUSSION

In this study, we investigated niche segregation and resource partitioning between brown bears and wolves within the home range (i.e., third order of selection; Johnson, 1980) in a long‐established protected area where both species have always coexisted and live at equilibrium densities. Our findings confirm that Apennine bears and wolves in the PNALM do reciprocally affect their niches and habitat selection patterns. Yet, because both the absolute and the relative density of bears and wolves clearly affect the extent and direction of their interspecific interactions, our findings pertain to the specific ecological and anthropogenic conditions in the PNALM and may not apply at lower bear and/or wolf population densities. Nevertheless, our findings do contribute to the accumulating evidence that interspecific interactions at the intraguild level are relevant in shaping species' realized niches, resource selection, and spatial distribution (e.g., Darnell et al., 2014; Tannerfeldt et al., 2002).

Depending on the season, the interaction between Apennine bears and wolves in the PNALM, although through unobserved behavioral mechanisms, had variable effects, ranging from niche overlap to niche segregation, as indicated by the K‐select analysis. In particular, the two species segregated their realized niches especially in autumn and, limited to daylight hours, during the spring, while they overlapped their niches during the other seasons. While this partially supports our H1, the same lack of segregation in early and late summer in turn support H2, related to the overriding effect of human activity on the interaction between these two large carnivore species. In addition, as revealed by multi‐species RSFs, fine‐scale resource selection further disclosed direction and extent of the interaction between the two species, indicating that both synergistic and antagonistic interactions may take place also if no segregation in the realized niche is apparent.

As revealed by K‐select, failure to detect niche segregation between bears and wolves in spring, except daylight hours, could be accounted for by several factors. First, competition between bears and wolves may be at its annual lowest, as bears emerge from their den by mid‐March in our study area and for about 2 weeks display lower movement and foraging rates (i.e., they are in a “walking hibernation” state; sensu Nelson et al., 1983). Second, overwinter ungulate carcasses and, successively, newborn ungulates that are particularly vulnerable to wolf predation are more available in this period, likely reducing competition between the two species (Lewis & Lafferty, 2014; Moleón et al., 2015), similarly to what has been reported for bear‐lynx interactions (Elbroch et al., 2015). Although Apennine bears are not particularly apt to predate wild ungulates, they easily relocate wolf kills and livestock carcasses left on the ground (Ciucci et al., 2014); coupled with this, the enhanced accessibility of prey and carcasses in this period might allow wolves to compensate for interference by bears (i.e., kleptoparasitism) through complementary responses in hunting activity. Third, adult male bears, those expectedly more competitive with wolves at wolf kills (Milleret et al., 2018), experience the peak in reproductive activity toward the end of the spring, and their interest in locating and usurping wolf kills might be lower and less persistent. Bears and wolves, however, segregated their niches in spring limited to daylight hours. Terrain ruggedness and high tree density of both beech and oak forests, on one hand, and increased distances from settlements and roads, forest edges, and open pastures, on the other hand, appeared to be proportionally more important for bears than wolves (Figure 2). This is likely an indication that, while both species tend to select the same habitat features to reduce human disturbance, wolves in spring are concerned of human disturbance to a lesser extent than bears; the low levels of human activity in this season, therefore, might allow wolves to reduce encounters with bears when selecting diurnal retreat areas. This is further supported by the lack of niche segregation in summer also during daylight hours, when the level of human activity markedly increases in the PNALM (Donatelli et al., 2022; Mancinelli et al., 2019). During summer months, activities such as forestry, livestock grazing, resource harvesting, and tourism (hiking, biking, horseback riding) intensify and extend also in the most remote areas of the park. Niche overlap between bears and wolves both in early and late summer might therefore be an indication of a convergent, risk‐aversion adaptation that translates into similar habitat requirements and a reduction of niche partitioning (Smith et al., 2017, 2018). In these seasons, the opportunity for bears and wolves in the PNALM to segregate their niches is limited due to the increased human encroachment, as indicated by their convergent selection for rugged terrain, densely forested areas, and increased distances from settlements and roads, the latter especially during late summer and with little variation throughout the day (Figure 2b,c). During fall, bears and wolves clearly segregated their niches. Not only in this season there occurs a marked reduction in human pressure but bears, in particular, are at the peak of their hyperphagic period and tend to ensure protracted and undisturbed accessibility to their seasonal key foods (in the PNALM, hard mast, and fleshy fruits; Ciucci et al., 2014); they therefore select for densely forested oak forests further from secondary roads and in rugged terrain (Figure 2d). This pattern was much less pronounced in wolves (Figure 2d), whose offspring are by now of larger size and, being able to travel with the rest of the pack, are less susceptible to human (and bear) disturbance.

We caution, however, that our K‐select analysis comes with two caveats. First, our failure to detect significant niche segregation between bears and wolves in early and late summer could be due to a relatively small sample size and hence a low power of the randomization test. Second, in our analysis we failed to account for different age and social categories that are known, both in bears (Milleret et al., 2018) and wolves (Mancinelli et al., 2019), to differ in habitat use and requirements.

Notably, by means of RSFs we detected resource partitioning between bears and wolves also in the seasons (i.e., spring and summer) when their realized niches overlapped. As indicated by the interspecific effect in multi‐species RSFs, both species reciprocally affected their habitat selection patterns, revealing both synergistic (for bears only) and antagonistic (for both bears and wolves) interactions. For instance, in spring we revealed differences in habitat selection between bears and wolves but no interspecific effects, likely indicating that interspecific competition is effectively reduced by resource partitioning due to marked ecological and behavioral differences (see 4. Discussion above for K‐select in spring). Differently, both in early and late summer, while K‐select did not detect niche segregation, multi‐species RSFs revealed that habitat use by both species reciprocally affected their habitat selection patterns. This suggests that under given conditions (e.g., increased human encroachment), niche segregation might not be a viable option, so that bears and wolves end up reciprocally affecting their habitat selection to prevent or reduce their interactions (i.e., fine‐scale resource partitioning). In early summer, for instance, the probability of resource selection by bears increased at higher selection values for wolves, likely responding to an enhanced opportunity to usurp wolf kills (Tallian et al., 2017). This tendency, however, reversed in late summer when wolves, attending and defending pups at rendezvous sites, are keener in chasing away intruding bears attracted by food scraps (Ballard et al., 2003; Stahler et al., 2020). The large amount of time that wolves spend at rendezvous sites, the presence of vulnerable pups, and the presence of prey remains make these areas hotspots for interspecific interactions (Ballard et al., 2003). Indeed, rendezvous sites expose vulnerable pups to a greater risk of predation, compared to den sites, because they are visible and more mobile (Ausband et al., 2016; Ruprecht et al., 2012). Kleptoparasitism by bears may be quite costly for wolves attending pups at rendezvous sites in late summer, a period during which the continued accessibility to wolf kills is particularly important (Ordiz et al., 2015). Bears, on the other hand, may be negatively affected by interference competition by wolf packs in their attempt to limit bears' occurrence in the proximity of rendezvous sites. Globally, interactions with bears by wolves at rendezvous sites have been observed less frequently compared to kill sites, but they still represent a noticeable proportion of documented interactions, playing a relevant role in bear‐wolf interaction dynamics (Ballard et al., 2003; Gunther & Smith, 2004; Stahler et al., 2020). The likelihood of antagonistic interactions between bears and wolves in our study area might increase in late summer because of the risk‐avoidance strategy to reduce impact by humans that forces wolves and bears to use retreat areas featuring similar habitat conditions. During fall, when bears are at the peak of the hyperphagic period, they may increasingly act as effective competitors for wolf kills, forcing wolves to select areas with lower chances of interference by bears. In the PNALM, convergent habitat use by bears and wolves wanes during fall likely in response to decreased anthropogenic pressure, translating in niche segregation and asymmetrical interactions, mostly accounting for the active avoidance of bears by wolves but not vice versa. Similar to K‐select, our RSFs‐related findings are therefore in line with H1, possibly due to the effect of kleptoparasitism by bears on wolves. Although kleptoparasitism by bears might not be sufficient to determine segregation by wolves, it may still exert costs to wolves while benefitting bears (Ballard et al., 2003; Milleret et al., 2018; Ordiz et al., 2015; Tallian et al., 2017). Although we do not have extensive observations of events of kleptoparasitism by bears of wolf kills in the PNALM, we reported through camera trapping some anecdotal cases of adult male bears defending large carrions by wolves. Brown bears are known to represent the primary, dominant scavenger within the guild of large carnivores in the northern hemisphere (Ballard et al., 2003; Krofel et al., 2012), an effect that is most prominent where bears live at high densities (Krofel & Jerina, 2016; Krofel et al., 2012), such as in our study area. These results are consistent with previous studies elsewhere, in which wolves appear negatively affected by the presence of bears (Ballard et al., 2003; Gunther & Smith, 2004; Milleret et al., 2018; Ordiz et al., 2015; Smith et al., 2003; Tallian et al., 2017). These negative effects on wolves may have implications at the population level in terms of distribution and habitat use (Milleret et al., 2018; Ordiz et al., 2015; Ordiz, Milleret et al., 2020; Ordiz, Uzal et al., 2020), predation rate, and even fitness (Tallian et al., 2017). In turn, these effects may have cascading effects that affect prey populations and other trophic levels (Berger et al., 2008; Elbroch et al., 2015; Kortello et al., 2007; Tallian et al., 2017). In our study area, however, the availability of anthropogenic subsidies in the form of large livestock carrions (i.e., horses, cattle) abandoned on the ground (Ciucci et al., 2020) may buffer the expectedly negative effects of kleptoparasitism by bears on the wolf population (i.e., reproduction and fitness), adding complexity to the dynamics of intraguild interactions and their cascading effects in human‐modified landscapes.

Our findings, and especially niche overlap between bears and wolves during summer months, suggest that anthropogenic pressure can potentially exacerbate the outcome of bear‐wolf interactions, increasing antagonistic interactions between the two species where and when they have no possibility to segregate. Similar effects were also documented in less densely populated European countries (Milleret et al., 2018; Ordiz et al., 2015), and may be particularly relevant in the PNALM due to its relatively small size. Here, although bears and wolves live at relatively high densities (Ciucci et al., 2015; Mancinelli et al., 2018), we obtained no indication that interspecific competition between them negatively affect one or both species. Local conditions may account for reduced level of competition, among which a reduced carnivory in Apennine bears (Ciucci et al., 2014) and the accessibility of large livestock carcasses abandoned on the ground (i.e., anthropogenic subsidies). Given the precarious conservation status of the Apennine brown bear (Benazzo et al., 2017), the interaction with wolves may be actually beneficial for bears, as the increased availability of wolf kills throughout the year might enhance their nutritional conditions and buffer against seasonal and annual fluctuations in other bear key foods. This emphasizes how an increased species diversity of large carnivores, and hence intraguild interactions, may be important to foster recovery of small and isolated bear populations where bears are the dominant scavenger (Servheen & Knight, 1993). Although population effects of such interactions are difficult to assess (Ordiz et al., 2015), it would be interesting for future investigations to evaluate if recovery of small and endangered bear populations will be facilitated by wolf occurrence at wider scales of analysis.

## AUTHOR CONTRIBUTIONS

**Cecilia Parracciani:** Conceptualization (equal); data curation (equal); formal analysis (lead); software (equal); writing – original draft (equal). **Luigi Maiorano:** Conceptualization (equal); formal analysis (equal); software (supporting); writing – review and editing (supporting). **Paolo Ciucci:** Conceptualization (equal); data curation (equal); formal analysis (supporting); funding acquisition (lead); investigation (lead); project administration (lead); supervision (lead); writing – original draft (equal); writing – review and editing (equal).

## FUNDING INFORMATION

This research project was funded by the Wildlife Conservation Society. Additional funding was provided by the European Union – NextGenerationEU National Biodiversity Future Center.

## CONFLICT OF INTEREST STATEMENT

The authors declare no competing interests.

## Data Availability

Data are deposited in the Dryad data repository: doi:10.5061/dryad.cjsxksndn available at this link: https://datadryad.org/stash/share/yGZtdSVJuQ87hSoes5GYTMQ8I2EcE6edic2zCIUSSdw.

## APPENDIX 1

**TABLE A1:** Study period and Global Positioning System (GPS) locations collected from adult Apennine brown bears (*n* = 19) and wolves (*n* = 7) to assess niche overlap and the effect of their interactions on habitat selection in the Abruzzo Lazio and Molise National Park, central Italy (2005–2010).

Species	ID (pack)^a^	Tracking period		GPS locations (no.)
		From	To	
Bear	F01	19‐May‐06	19‐Nov‐06	1141
	F02_2005	28‐Oct‐05	4‐Dec‐05	239
	F02_2006	22‐Mar‐06	9‐Nov‐06	1411
	F03	12‐Jul‐06	28‐Apr‐07	440
	F04	9‐Aug‐06	11‐Sep‐06	97
	F05	6‐Sep‐06	19‐Jul‐07	394
	F06	8‐Nov‐06	26‐May‐07	176
	F07_2007	21‐May‐07	8‐Dec‐07	440
	F07_2008	14‐Jun‐08	10‐Nov‐08	463
	F08_2008	1‐Nov‐08	8‐Dec‐08	136
	F08_2009	26‐Mar‐09	10‐Nov‐09	648
	F08_2010	19‐Apr‐10	24‐May‐10	132
	F09	10‐Sep‐09	19‐Oct‐09	158
	F10_2008	1‐Nov‐08	10‐Nov‐08	80
	F10_2009	19‐Apr‐09	29‐Oct‐09	237
	F10_2010	6‐Apr‐10	12‐Nov‐10	568
	F13_2009	20‐Oct‐09	29‐Oct‐09	78
	F13_2010	11‐Apr‐10	16‐Nov‐10	566
	M01_2005	7‐Jul‐05	28‐Aug‐05	268
	M01_2006	25‐May‐06	14‐Aug‐06	462
	M01_2009	17‐May‐09	15‐Sep‐09	304
	M02	19‐Oct‐05	19‐Dec‐05	416
	M04	9‐Aug‐06	6‐Sep‐06	82
	M06	19‐Apr‐07	15‐Sep‐07	329
	M07	24‐Apr‐07	23‐Jun‐07	152
	M08	9‐Aug‐07	13‐Oct‐07	199
	M10_2008	14‐Jun‐08	10‐Nov‐08	387
	M10_2009	10‐Aug‐09	20‐Oct‐09	167
	M11	14‐Jun‐08	8‐Sep‐08	195
Wolf	F23 (Canneto)	5‐Nov‐09	10‐Jul‐10	330
	F24 (Bisegna)	1‐Mar‐10	16‐Jun‐10	461
	F24 (Villa)	13‐May‐09	31‐Dec‐09	652
	F25 (Orsara)	1‐Nov‐09	19‐May‐10	701
	M26 (Canneto)	1‐Nov‐09	26‐Aug‐10	1508
	M27 (Iorio)	17‐Nov‐09	31‐Dec‐09	232
	M28 (Collelongo)	25‐May‐10	21‐Oct‐10	880
	M30 (Orsara)	30‐Oct‐10	31‐Dec‐10	173

*Note*: GPS locations were standardized to a common acquisition rate of 1 relocation/3 h.

^a^ F, female; M, male.

**TABLE A2:** Multivariate correlation, measured by the Variance Inflation Factor (VIF), among covariates used to develop seasonal, multi‐species Resource Selection Functions to investigate third‐order habitat selection by Apennine brown bears and wolves in the Abruzzo Lazio and Molise National Park, central Italy (2005–2010).

Species	Variable	VIF – spring	VIF – early summer	VIF – late summer	VIF – autumn
Bear	Agri	1.69	1.8	1.88	1.98
	NoVeg	3.04	2.48	2.63	6.91
	PastGrass	3.68	3.83	3.89	4.03
	Shrub	1.61	1.49	1.5	1.29
	DistForEdge	3.87	3.69	3.01	4.25
	DistSettlR1	2.43	2.31	2.12	1.93
	DistR2	2.54	1.92	2.13	2.83
	Hillsh	1.13	1.16	1.3	1.23
	TRI	1.39	2.25	1.64	1.91
	TDBeech	5.52	5.83	5.58	5.86
	TDOak	4.03	4.13	3.65	3.15
	WolfRSF	4.27	3.59	2.47	9.01
Wolf	Agri	2.29	2.55	2.46	2.34
	NoVeg	2.42	2.26	3.51	2.18
	PastGrass	3.35	2.74	2.79	3.65
	Shrub	1.32	1.35	1.43	1.58
	DistForEdge	2.71	2.58	2.81	2.74
	DistSettlR1	2.2	2.6	1.67	2.59
	DistR2	1.93	1.72	2.44	2.52
	Hillsh	1.16	1.27	1.09	1.34
	TRI	2.73	1.96	1.42	3.06
	TDBeech	3.27	3.22	4.63	3.73
	TDOak	2.32	2.75	2.83	3.03
	BearRSF	5.13	5.53	5.32	8.41

**TABLE A3:** Model selection for seasonal, multi‐species resource selection functions to assess the effect of the interaction with wolves on third‐order habitat selection by Apennine brown bears in the Abruzzo Lazio and Molise National Park, central Itay (2005–2010).

Season	Model	Description	*K*	AIC_*c*_	ΔAIC_*c*_	*w*_*i*_
Spring	1	Agri + DistForEdge + DistR2 + DistSettlR1 + Hillsh + NoVeg + PastGrass + Shrub + TDBeech + TDOak + TRI	13	10634.97	0.00	0.28
	2	Agri + DistForEdge + DistR2 + DistSettlR1 + Hillsh + NoVeg + PastGrass + TDBeech + TDOak + TRI	12	10635.74	0.77	0.19
	3	Agri + DistForEdge + DistR2 + DistSettlR1 + Hillsh + NoVeg + PastGrass + WolfRSF + Shrub + TDBeech + TDOak + TRI	14	10636.64	1.67	0.12
	4	Agri + DistForEdge + DistR2 + DistSettlR1 + Hillsh + NoVeg + PastGrass + Shrub + TDBeech + TRI	12	10636.69	1.72	0.12
	5	Agri + DistForEdge + DistR2 + DistSettlR1 + Hillsh + NoVeg + PastGrass + WolfRSF + TDBeech + TDOak + TRI	13	10636.73	1.76	0.11
	6	Agri + DistForEdge + DistR2 + DistSettlR1 + Hillsh + NoVeg + PastGrass + WolfRSF + Shrub + TDBeech + TRI	13	10638.50	3.53	0.05
	7	Agri + DistForEdge + DistR2 + Hillsh + NoVeg + PastGrass + WolfRSF + TDBeech + TDOak + TRI	12	10639.02	4.05	0.04
	8	Agri + DistForEdge + DistR2 + Hillsh + NoVeg + PastGrass + WolfRSF + Shrub + TDBeech + TDOak + TRI	13	10639.08	4.11	0.04
	9	Agri + DistForEdge + DistR2 + DistSettlR1 + Hillsh + NoVeg + PastGrass + TDBeech + TRI	11	10640.77	5.80	0.02
	10	Agri + DistForEdge + DistR2 + Hillsh + NoVeg + PastGrass + WolfRSF + Shrub + TDBeech + TRI	12	10641.42	6.45	0.01
	11	Agri + DistForEdge + DistR2 + DistSettlR1 + Hillsh + NoVeg + PastGrass + WolfRSF + TDBeech + TRI	12	10641.61	6.63	0.01
	12	DistForEdge + DistR2 + Hillsh + NoVeg + PastGrass + WolfRSF + TDBeech + TDOak + TRI	11	10642.94	7.97	0.01
	13	DistForEdge + DistR2 + DistSettlR1 + Hillsh + NoVeg + PastGrass + WolfRSF + TDBeech + TDOak + TRI	12	10643.06	8.08	0.00
	14	Agri + DistForEdge + DistR2 + Hillsh + NoVeg + PastGrass + Shrub + TDBeech + TDOak + TRI	12	10643.13	8.16	0.00
	15	DistForEdge + DistR2 + DistSettlR1 + Hillsh + NoVeg + PastGrass + TDBeech + TDOak + TRI	11	10643.21	8.24	0.00
Early Summer	1	Agri + DistForEdge + DistR2 + Hillsh + NoVeg + PastGrass + WolfRSF + TDBeech + TDOak + TRI	12	15758.48	0.00	0.15
	2	Agri + DistForEdge + DistR2 + Hillsh + NoVeg + WolfRSF + Shrub + TDBeech + TDOak + TRI	12	15758.52	0.05	0.14
	3	Agri + DistForEdge + DistR2 + Hillsh + NoVeg + PastGrass + WolfRSF + Shrub + TDBeech + TDOak + TRI	13	15758.55	0.08	0.14
	4	Agri + DistForEdge + DistR2 + DistSettlR1 + Hillsh + NoVeg + PastGrass + WolfRSF + TDBeech + TDOak + TRI	13	15758.80	0.32	0.12
	5	Agri + DistForEdge + DistR2 + DistSettlR1 + Hillsh + NoVeg + PastGrass + WolfRSF + Shrub + TDBeech + TDOak + TRI	14	15759.52	1.04	0.09
	6	Agri + DistForEdge + Hillsh + NoVeg + WolfRSF + Shrub + TDBeech + TDOak + TRI	11	15759.98	1.50	0.07
	7	Agri + DistForEdge + DistR2 + DistSettlR1 + Hillsh + NoVeg + WolfRSF + Shrub + TDBeech + TDOak + TRI	13	15760.05	1.58	0.07
	8	Agri + DistForEdge + Hillsh + NoVeg + PastGrass + WolfRSF + Shrub + TDBeech + TDOak + TRI	12	15760.75	2.27	0.05
	9	Agri + DistForEdge + DistR2 + Hillsh + NoVeg + WolfRSF + TDBeech + TDOak + TRI	11	15760.92	2.45	0.04
	10	Agri + DistForEdge + Hillsh + NoVeg + PastGrass + WolfRSF + TDBeech + TDOak + TRI	11	15761.47	2.99	0.03
	11	Agri + DistForEdge + DistSettlR1 + Hillsh + NoVeg + WolfRSF + TDBeech + TDOak + TRI	12	15761.92	3.44	0.03
	12	Agri + DistForEdge + DistR2 + DistSettlR1 + Hillsh + NoVeg + WolfRSF + TDBeech + TDOak + TRI	12	15761.99	3.51	0.03
	13	Agri + DistForEdge + DistSettlR1 + Hillsh + NoVeg + PastGrass + WolfRSF + Shrub + TDBeech + TDOak + TRI	13	15762.54	4.07	0.02
	14	Agri + DistForEdge + DistSettlR1 + Hillsh + NoVeg + PastGrass + WolfRSF + TDBeech + TDOak + TRI	12	15762.92	4.45	0.02
	15	Agri + DistForEdge + Hillsh + NoVeg + WolfRSF + TDBeech + TDOak + TRI	10	15762.95	4.47	0.02
Late Summer	1	Agri + DistForEdge + DistR2 + DistSettlR1 + NoVeg + PastGrass + WolfRSF + Shrub + TDBeech + TDOak + TRI	13	16837.39	0.00	0.24
	2	DistForEdge + DistR2 + DistSettlR1 + NoVeg + PastGrass + WolfRSF + Shrub + TDBeech + TDOak + TRI	12	16837.46	0.07	0.23
	3	Agri + DistForEdge + DistR2 + DistSettlR1 + Hillsh + NoVeg + PastGrass + WolfRSF + Shrub + TDBeech + TDOak + TRI	14	16838.29	0.90	0.15
	4	DistForEdge + DistR2 + DistSettlR1 + Hillsh + NoVeg + PastGrass + WolfRSF + Shrub + TDBeech + TDOak + TRI	13	16838.31	0.92	0.15
	5	Agri + DistR2 + DistSettlR1 + NoVeg + PastGrass + WolfRSF + Shrub + TDBeech + TDOak + TRI	12	16839.75	2.36	0.07
	6	DistR2 + DistSettlR1 + NoVeg + PastGrass + WolfRSF + Shrub + TDBeech + TDOak + TRI	11	16840.71	3.32	0.05
	7	Agri + DistR2 + DistSettlR1 + Hillsh + NoVeg + PastGrass + WolfRSF + Shrub + TDBeech + TDOak + TRI	13	16840.88	3.49	0.04
	8	DistR2 + DistSettlR1 + Hillsh + NoVeg + PastGrass + WolfRSF + Shrub + TDBeech + TDOak + TRI	12	16841.81	4.42	0.03
	9	Agri + DistForEdge + DistR2 + DistSettlR1 + NoVeg + PastGrass + WolfRSF + Shrub + TDBeech + TDOak + TRI	12	16843.51	6.12	0.01
	10	DistForEdge + DistR2 + DistSettlR1 + NoVeg + PastGrass + WolfRSF + Shrub + TDBeech + TDOak	11	16843.84	6.45	0.01
	11	Agri + DistR2 + DistSettlR1 + NoVeg + PastGrass + WolfRSF + Shrub + TDBeech + TDOak	11	16845.17	7.78	0.00
	12	Agri + DistForEdge + DistR2 + DistSettlR1 + Hillsh + NoVeg + PastGrass + WolfRSF + Shrub + TDBeech + TDOak	13	16845.24	7.85	0.00
	13	DistForEdge + DistR2 + DistSettlR1 + Hillsh + NoVeg + PastGrass + WolfRSF + Shrub + TDBeech + TDOak	12	16845.56	8.17	0.00
	14	DistR2 + DistSettlR1 + NoVeg + PastGrass + WolfRSF + Shrub + TDBeech + TDOak	10	16846.36	8.97	0.00
	15	Agri + DistR2 + DistSettlR1 + Hillsh + NoVeg + PastGrass + WolfRSF + Shrub + TDBeech + TDOak	12	16846.98	9.59	0.00
Autumn	1	Agri + DistR2 + DistSettlR1 + Hillsh + NoVeg + PastGrass + WolfRSF + Shrub + TDBeech + TDOak + TRI	13	14814.64	0.00	0.21
	2	Agri + DistForEdge + DistR2 + DistSettlR1 + Hillsh + NoVeg + PastGrass + WolfRSF + Shrub + TDBeech + TDOak + TRI	14	14815.53	0.89	0.13
	3	Agri + DistR2 + DistSettlR1 + Hillsh + PastGrass + WolfRSF + Shrub + TDBeech + TDOak + TRI	12	14815.60	0.96	0.13
	4	Agri + DistR2 + DistSettlR1 + Hillsh + PastGrass + Shrub + TDBeech + TDOak + TRI	11	14816.75	2.11	0.07
	5	Agri + DistR2 + DistSettlR1 + Hillsh + PastGrass + WolfRSF + Shrub + TDOak + TRI	11	14816.91	2.27	0.07
	6	Agri + DistForEdge + DistR2 + DistSettlR1 + Hillsh + PastGrass + WolfRSF + Shrub + TDBeech + TDOak + TRI	13	14817.19	2.55	0.06
	7	Agri + DistR2 + DistSettlR1 + NoVeg + PastGrass + WolfRSF + Shrub + TDBeech + TDOak + TRI	12	14818.25	3.62	0.03
	8	Agri + DistForEdge + DistR2 + DistSettlR1 + Hillsh + PastGrass + Shrub + TDBeech + TDOak + TRI	12	14818.35	3.71	0.03
	9	Agri + DistForEdge + DistR2 + DistSettlR1 + NoVeg + PastGrass + WolfRSF + Shrub + TDBeech + TDOak + TRI	13	14818.35	3.71	0.03
	10	Agri + DistR2 + DistSettlR1 + Hillsh + NoVeg + PastGrass + Shrub + TDBeech + TDOak + TRI	12	14818.42	3.79	0.03
	11	Agri + DistForEdge + DistR2 + DistSettlR1 + Hillsh + PastGrass + Shrub + TDOak + TRI	11	14818.63	4.00	0.03
	12	Agri + DistR2 + DistSettlR1 + Hillsh + NoVeg + PastGrass + WolfRSF + Shrub + TDOak + TRI	12	14818.69	4.05	0.03
	13	Agri + DistR2 + DistSettlR1 + Hillsh + PastGrass + Shrub + TDOak + TRI	10	14818.77	4.13	0.03
	14	Agri + DistForEdge + DistR2 + DistSettlR1 + Hillsh + PastGrass + WolfRSF + Shrub + TDOak + TRI	12	14818.81	4.17	0.03
	15	Agri + DistR2 + DistSettlR1 + Hillsh + NoVeg + PastGrass + Shrub + TDOak + TRI	11	14819.04	4.40	0.02

*Note*: *K* = number of parameters; AIC_*c*_ = Akaike's Information Criterion adjusted for small sample size; *w*_*i*_ = Akaike weights. Only models with ΔAIC_c_ < 10 are shown.

**TABLE A4:** Model selection for seasonal, multi‐species resource selection functions to assess the effect of the interaction with Apennine brown bears on third‐order habitat selection by wolves in the Abruzzo Lazio and Molise National Park, central Itay (2005–2010).

Season	Model	Description	*K*	AIC_*c*_	ΔAIC_*c*_	*w*_*i*_
Spring	1	DistForedge + DistR2 + DistSettlR1 + Hillsh + NoVeg + Shrub + TDBeech	9	7858.34	0.00	0.05
	2	DistForedge + DistR2 + DistSettlR1 + Hillsh + NoVeg + Shrub + TDBeech + TDOak	10	7858.59	0.25	0.05
	3	DistForedge + DistR2 + DistSettlR1 + Hillsh + NoVeg + TDBeech	8	7859.06	0.71	0.04
	4	Agri + DistForedge + DistR2 + DistSettlR1 + Hillsh + NoVeg + Shrub + TDBeech	10	7859.11	0.77	0.04
	5	DistForedge + DistR2 + DistSettlR1 + Hillsh + NoVeg + TDBeech + TDOak	9	7859.16	0.81	0.04
	6	DistForedge + DistR2 + DistSettlR1 + Hillsh + NoVeg + Shrub + TDBeech + TDOak + TRI	11	7859.75	1.40	0.03
	7	DistForedge + DistR2 + DistSettlR1 + Hillsh + NoVeg + TDBeech + TDOak + TRI	10	7859.96	1.62	0.02
	8	Agri + DistForedge + DistR2 + DistSettlR1 + Hillsh + NoVeg + Shrub + TDBeech + TDOak	11	7860.05	1.71	0.02
	9	DistForedge + DistR2 + DistSettlR1 + Hillsh + NoVeg + Shrub + TDBeech + TRI	10	7860.13	1.79	0.02
	10	DistForedge + DistR2 + DistSettlR1 + Hillsh + NoVeg + BearRSF + Shrub + TDBeech	10	7860.17	1.83	0.02
	11	Agri + DistForedge + DistR2 + DistSettlR1 + Hillsh + NoVeg + TDBeech	9	7860.32	1.97	0.02
	12	DistForedge + DistR2 + DistSettlR1 + Hillsh + NoVeg + PastGrass + Shrub + TDBeech	10	7860.34	1.99	0.02
	13	DistForedge + DistR2 + DistSettlR1 + Hillsh + NoVeg + PastGrass + Shrub + TDBeech + TDOak	11	7860.50	2.16	0.02
	14	DistForedge + DistR2 + DistSettlR1 + Hillsh + NoVeg + BearRSF + Shrub + TDBeech + TDOak	11	7860.59	2.24	0.02
	15	DistForedge + DistR2 + DistSettlR1 + Hillsh + NoVeg + TDBeech + TRI	9	7860.67	2.33	0.02
Early Summer	1	Agri + DistForedge + DistR2 + DistSettlR1 + Hillsh + NoVeg + PastGrass + BearRSF + Shrub + TDBeech + TRI	13	5709.63	0.00	0.52
	2	Agri + DistForedge + DistR2 + DistSettlR1 + Hillsh + NoVeg + PastGrass + BearRSF + Shrub + TDBeech + TDOak + TRI	14	5711.48	1.85	0.21
	3	Agri + DistForedge + DistR2 + DistSettlR1 + Hillsh + NoVeg + PastGrass + Shrub + TDBeech + TRI	12	5713.62	3.99	0.07
	4	Agri + DistForedge + DistR2 + DistSettlR1 + Hillsh + NoVeg + PastGrass + Shrub + TDBeech + TDOak + TRI	13	5714.25	4.62	0.05
	5	DistForedge + DistR2 + DistSettlR1 + Hillsh + NoVeg + PastGrass + BearRSF + Shrub + TDBeech + TRI	12	5715.30	5.67	0.03
	6	Agri + DistForedge + DistR2 + DistSettlR1 + Hillsh + NoVeg + BearRSF + Shrub + TDBeech + TRI	12	5715.32	5.68	0.03
	7	DistForedge + DistR2 + DistSettlR1 + Hillsh + NoVeg + PastGrass + BearRSF + Shrub + TDBeech + TDOak + TRI	13	5715.76	6.13	0.02
	8	Agri + DistForedge + DistR2 + DistSettlR1 + Hillsh + NoVeg + BearRSF + Shrub + TDBeech + TDOak + TRI	13	5716.74	7.10	0.01
	9	Agri + DistForedge + DistR2 + DistSettlR1 + Hillsh + NoVeg + PastGrass + BearRSF + Shrub + TDOak + TRI	13	5716.90	7.27	0.01
	10	DistForedge + DistR2 + DistSettlR1 + Hillsh + NoVeg + Shrub + TDBeech + TRI	11	5717.33	7.70	0.01
	11	Agri + DistForedge + DistR2 + DistSettlR1 + Hillsh + NoVeg + PastGrass + BearRSF + Shrub + TRI	12	5717.48	7.85	0.01
	12	DistForedge + DistR2 + DistSettlR1 + Hillsh + NoVeg + BearRSF + Shrub + TDBeech + TDOak + TRI	12	5717.71	8.08	0.01
	13	DistForedge + DistR2 + DistSettlR1 + Hillsh + NoVeg + PastGrass + Shrub + TDBeech + TDOak + TRI	12	5718.39	8.76	0.01
	14	Agri + DistForedge + DistR2 + DistSettlR1 + Hillsh + NoVeg + PastGrass + Shrub + TRI	11	5719.39	9.75	0.00
Late Summer	1	DistForedge + DistR2 + DistSettlR1 + Hillsh + NoVeg + PastGrass + BearRSF + Shrub + TDBeech + TDOak + TRI	13	4199.56	0.00	0.50
	2	Agri + DistForedge + DistR2 + DistSettlR1 + Hillsh + NoVeg + PastGrass + BearRSF + Shrub + TDBeech + TDOak + TRI	14	4200.61	1.05	0.30
	3	Agri + DistForedge + DistR2 + DistSettlR1 + Hillsh + NoVeg + PastGrass + BearRSF + TDBeech + TDOak + TRI	13	4202.92	3.36	0.09
	4	DistForedge + DistR2 + DistSettlR1 + Hillsh + NoVeg + PastGrass + BearRSF + TDBeech + TDOak + TRI	12	4203.45	3.89	0.07
	5	DistForedge + DistR2 + DistSettlR1 + Hillsh + NoVeg + PastGrass + BearRSF + Shrub + TDBeech + TRI	12	4205.28	5.72	0.03
	6	Agri + DistForedge + DistR2 + DistSettlR1 + Hillsh + NoVeg + PastGrass + BearRSF + Shrub + TDBeech + TRI	13	4207.25	7.69	0.01
Autumn	1	Agri + DistForedge + DistR2 + Hillsh + NoVeg + PastGrass + BearRSF + TDOak	10	9547.08	0.00	0.21
	2	Agri + DistForedge + DistR2 + Hillsh + NoVeg + PastGrass + BearRSF + TDBeech + TDOak	11	9548.16	1.07	0.13
	3	Agri + DistForedge + DistR2 + DistSettlR1 + Hillsh + NoVeg + PastGrass + BearRSF + TDOak	11	9548.59	1.51	0.10
	4	Agri + DistForedge + DistR2 + Hillsh + NoVeg + PastGrass + BearRSF + Shrub + TDOak	11	9548.71	1.62	0.10
	5	Agri + DistForedge + DistR2 + Hillsh + NoVeg + PastGrass + BearRSF + TDOak + TRI	11	9549.07	1.98	0.08
	6	Agri + DistForedge + DistR2 + DistSettlR1 + Hillsh + NoVeg + PastGrass + BearRSF + TDBeech + TDOak	12	9549.53	2.44	0.06
	7	Agri + DistForedge + DistR2 + Hillsh + NoVeg + PastGrass + BearRSF + Shrub + TDBeech + TDOak	12	9550.03	2.95	0.05
	8	Agri + DistForedge + DistR2 + DistSettlR1 + Hillsh + NoVeg + PastGrass + BearRSF + Shrub + TDOak	12	9550.04	2.96	0.05
	9	Agri + DistForedge + DistR2 + Hillsh + NoVeg + PastGrass + BearRSF + TDBeech + TDOak + TRI	12	9550.10	3.02	0.05
	10	Agri + DistForedge + DistR2 + DistSettlR1 + Hillsh + NoVeg + PastGrass + BearRSF + TDOak + TRI	12	9550.44	3.35	0.04
	11	Agri + DistForedge + DistR2 + Hillsh + NoVeg + PastGrass + BearRSF + Shrub + TDOak + TRI	12	9550.65	3.56	0.04
	12	Agri + DistForedge + DistR2 + DistSettlR1 + Hillsh + NoVeg + PastGrass + BearRSF + Shrub + TDBeech + TDOak	13	9551.30	4.21	0.03
	13	Agri + DistForedge + DistR2 + DistSettlR1 + Hillsh + NoVeg + PastGrass + BearRSF + TDBeech + TDOak + TRI	13	9551.53	4.45	0.02
	14	Agri + DistForedge + DistR2 + DistSettlR1 + Hillsh + NoVeg + PastGrass + BearRSF + Shrub + TDOak + TRI	13	9551.69	4.60	0.02
	15	Agri + DistForedge + DistR2 + Hillsh + NoVeg + PastGrass + BearRSF + Shrub + TDBeech + TDOak + TRI	13	9552.02	4.93	0.02

*Note*: *K* = number of parameters; AIC_*c*_ = Akaike's Information Criterion adjusted for small sample size; *w*_*i*_ = Akaike weights. Only models with ΔAIC_c_ < 10 are shown. See Table 1 for variable coding.

**TABLE A5:** Coefficients of seasonal Resource Selection Functions (RSF) to contrast third‐order habitat selection by Apennine brown bears in the Abruzzo Lazio and Molise National Park, central Italy (2005–2010), without (single species RSF) and with (multi‐species RSF) the interspecific wolf predictor (i.e., the probability of resource selection by wolves).

Variable	Spring						Early summer						Late summer						Autumn					
	Single species			Muti‐species			Single species			Muti‐species			Single species			Muti‐species			Single species			Muti‐species		
	β	95% CI		β	95% CI		β	95% CI		β	95% CI		β	95% CI		β	95% CI		β	95% CI		β	95% CI	
		Lower	Upper		Lower	Upper		Lower	Upper		Lower	Upper		Lower	Upper		Lower	Upper		Lower	Upper		Lower	Upper
Intercept	−2.96	−3.56	−2.37	−2.96	−3.5	−2.4	−2.59	−3.00	−2.19	−2.59	−2.98	−2.21	−2.52	−3.00	−2.04	−2.53	−2.99	−2.07	−2.31	−2.76	−1.86	−2.31	−2.74	−1.88
Agri	−.15	−0.27	−0.05	−.16	−0.28	−0.04	.19	0.12	0.26	.19	0.12	0.26	−.23	−0.43	−0.07	−.06	−0.23	0.10	.12	0.06	0.18	.17	0.11	0.23
NoVeg	−.35	−0.47	−0.24	−.46	−0.59	−0.33	.22	0.15	.29	0.29	0.22	0.35	.59	0.52	0.65	.49	0.42	0.56	−.07	−0.15	−0.00	.04	−0.09	0.17
PastGrass	−.70	−0.83	−0.57	−.75	−0.88	−0.61	−.12	−0.21	−0.02	−.05	−0.16	0.06	.38	0.29	0.48	.27	0.17	0.37	−.40	−0.49	−0.30	−.27	−0.38	−0.16
Shrub	−.03	−0.10	0.03	−.04	−0.12	0.04	.01	−0.05	0.07	.03	−0.04	0.10	.34	0.29	0.39	.28	0.23	0.33	−.13	−0.19	−0.07	−.09	−0.16	−0.03
DistForEdge	.19	0.09	0.28	.25	0.13	0.36	.07	−0.00	0.15	.17	0.08	0.25	−.05	−0.13	0.02	−.07	−0.16	0.03	−.05	−0.12	0.02	.01	−0.06	0.08
DistR2	.29	0.23	0.35	.28	0.20	0.37	.14	0.10	0.19	.05	−0.02	0.12	.31	0.26	0.35	.43	0.37	0.49	.22	0.17	0.27	.19	0.11	0.27
DistSettlR1	−.30	−0.38	−0.23	−.11	−0.21	−0.00	.21	0.15	0.27	.01	−0.05	0.08	−.13	−0.19	−0.07	.27	0.17	0.38	−.16	−0.23	−0.09	−.21	−0.29	−0.14
TDBeech	.30	0.19	0.42	.23	0.09	0.37	.44	0.33	0.54	.43	0.32	0.54	.75	0.64	0.86	.68	0.57	0.79	−.17	−0.27	−0.07	.08	−0.05	0.21
TDOak	.08	−0.01	0.18	.09	−0.04	0.21	.65	0.57	0.73	.65	0.56	0.74	.22	0.12	0.32	.23	0.13	0.33	.14	0.06	0.22	.28	0.20	0.35
Hillsh	−.12	−0.17	−0.07	−.12	−0.17	−0.07	−.13	−0.17	−0.09	−.16	−0.20	−0.12	−.07	−0.11	−0.02	.01	−0.03	0.05	−.07	−0.12	−0.03	−.06	−0.11	0.00
TRI	.36	0.31	0.42	.39	0.33	0.44	.21	0.16	0.26	.33	0.26	0.40	−.01	−0.06	0.04	−.08	−0.15	−0.02	.24	0.18	0.29	.22	0.15	0.29
WolfRSF	–	–	–	.02	−0.07	0.12	–	–	–	.23	0.15	0.32	–	–	–	−.26	−0.33	−0.19	–	–	–	.08	−0.07	0.23

*Note*: See Table 1 for variable coding.

**TABLE A6:** Coefficients of seasonal Resource Selection Functions (RSF) to contrast third‐order habitat selection by wolves in the Abruzzo Lazio and Molise National Park, central Italy (2005–2010), without (single species RSF) and with (multi‐species RSF) the interspecific bear predictor (i.e., the probability of resource selection by bears).

Variable	Spring						Early summer						Late summer						Autumn					
	Single species			Multi‐species			Single species			Multi‐species			Single species			Multi‐species			Single species			Multi‐species		
	β	95% CI		β	95% CI		β	95% CI		β	95% CI		β	95% CI		β	95% CI		β	95% CI		β	95% CI	
		Lower	Upper		Lower	Upper		Lower	Upper		Lower	Upper		Lower	Upper		Lower	Upper		Lower	Upper		Lower	Upper
Intercept	−2.26	−3.37	−1.17	−2.26	−3.14	−1.38	−2.09	−2.60	−1.56	−2.11	−2.49	−1.72	−2.56	−3.20	−1.93	−2.6	−3.15	−2.07	−1.62	−2.38	−0.86	−1.63	−2.24	−1.02
Agri	−.13	−0.23	−0.03	−.01	−0.08	0.05	−.29	−0.51	−0.10	−.23	−0.46	0.01	.05	−0.10	0.20	.03	−0.09	0.15	.02	−0.05	0.09	.19	0.12	0.26
NoVeg	−.73	−0.96	−0.53	−.67	−0.88	−0.45	−.15	−0.25	−0.04	−.19	−0.30	−0.08	−.16	−0.29	−0.04	.54	0.39	0.69	−.32	−0.41	−0.23	−.22	−0.30	−0.13
PastGrass	−.08	−0.18	0.03	.00	−0.05	0.05	−.24	−0.38	−0.10	−.18	−0.34	−0.01	−.38	−0.54	−0.22	−.34	−0.49	−0.19	−.31	−0.40	−0.21	−.23	−0.32	−0.15
Shrub	−.09	−0.18	−0.01	−.04	−0.12	0.05	−.28	−0.41	−0.15	−.27	−0.40	−0.14	−.24	−0.37	−0.11	−.13	−0.29	0.04	−.01	−0.07	0.05	.01	−0.03	0.04
DistForEdge	−.39	−0.48	−0.30	−.40	−0.48	−0.31	−.59	−0.69	−0.49	−.61	−0.72	−0.50	−.23	−0.36	−0.10	−.31	−0.43	−0.18	−.12	−0.20	−0.04	−.14	−0.22	−0.06
DistR2	.40	0.33	0.48	.38	0.30	0.45	.50	0.42	0.58	.47	0.38	0.57	.70	0.61	0.80	.99	0.85	1.12	.17	0.10	0.24	.28	0.20	0.36
DistSettlR1	−.41	−0.50	−0.31	−.44	−0.54	−0.34	.79	0.70	0.89	.74	0.62	0.86	.97	0.86	1.08	.84	0.73	0.95	.10	0.03	0.17	.01	−0.05	0.07
TDBeech	−.03	−0.16	0.10	.15	0.06	0.25	.15	−0.01	0.31	.19	0.04	0.34	.06	−0.13	0.25	.37	0.18	0.55	−.32	−0.44	−0.20	−.02	−0.08	0.05
TDOak	−.06	−0.15	0.03	.02	−0.05	0.09	.02	−0.11	0.15	.01	−0.07	0.09	.14	−0.02	0.29	.20	0.04	0.36	−.11	−0.19	−0.02	.29	0.21	0.37
Hills	.06	−0.01	0.11	.06	−0.01	0.13	.16	0.09	0.24	.20	0.11	0.28	.46	0.37	0.56	.43	0.33	0.53	−.03	−0.09	0.02	−.10	−0.15	−0.04
TRI	−.01	−0.08	0.06	−.02	−0.09	0.05	−.64	−0.73	−0.55	−.71	−0.83	−0.59	−.50	−0.60	−0.40	−.58	−0.69	−0.47	−.07	−0.14	−0.01	−.00	−0.05	0.04
BearRSF	–	–	–	.01	−0.06	0.08	–	–	–	.17	−0.03	0.37	–	–	–	−.70	−0.90	−0.51	–	–	–	−.27	−0.37	−0.16

*Note*: See Table 1 for variable coding.
